# Effect of insulin-like growth factor-1 complex of Simmental bull seminal plasma on post-thawed Kacang buck semen fertility

**DOI:** 10.14202/vetworld.2021.2073-2084

**Published:** 2021-08-11

**Authors:** Suherni Susilowati, Imam Mustofa, Wurlina Wurlina, Indah Norma Triana, Suzanita Utama, Rimayanti Rimayanti

**Affiliations:** 1 Laboratory of Veterinary Artificial Insemination, Division of Veterinary Reproduction, Faculty of Veterinary Medicine, Universitas Airlangga, Kampus C Unair, Mulyorejo, Surabaya, Indonesia; 2 Laboratory of Veterinary Obstetrics, Division of Veterinary Reproduction, Faculty of Veterinary Medicine, Universitas Airlangga, Kampus C Unair, Mulyorejo, Surabaya, Indonesia; 3 Laboratory of Veterinary Infertility and Sterility, Division of Veterinary Reproduction, Faculty of Veterinary Medicine, Universitas Airlangga, Kampus C Unair, Mulyorejo, Surabaya, Indonesia

**Keywords:** acrosome reaction, capacitation, good health and well-being, intact plasma membrane, motility, viability

## Abstract

**Background and Aim:**

Kacang buck sperm is cryosensitive due to the seminal plasma of semen itself. Meanwhile, bull seminal plasma contains the insulin-like growth factor-1 (IGF-1) complex, which is cryoprotective. The addition of the crude protein of Simmental bull seminal plasma increased the quality of post-thawed semen of Kacang buck. The study was conducted to determine the effects of Simmental bull seminal plasma with IGF-1 on the fertility of post-thawed Kacang buck semen.

**Materials and Methods:**

Buck semen was diluted in the following skim milk-egg yolk extender preparations: Without the addition of Simmental bull seminal plasma IGF-1 complex protein (T0); with the addition of 12-μg Simmental bull seminal plasma IGF-1 complex protein (T1); and with the addition of 24-μg Simmental bull seminal plasma IGF-1 complex protein (T2). The extended semen was packed in 0.25-mL straws and frozen. Post-thawed semen fertility was evaluated based on the following variables: Sperm motility, viability, intact plasma membrane (IPM), malondialdehyde (MDA) levels, capacitation status, and acrosome reaction. The difference in each variable among the groups was evaluated using analysis of variance, followed by Tukey’s honestly significant difference test, at a 95% level of significance. Meanwhile, principal component analysis (PCA) was used to identify the principal component of semen fertility among the seven parameters.

**Results:**

The T1 group showed the highest sperm motility, viability, IPM, and percentage of incapacitated sperm and the lowest MDA levels, percentage of capacitated sperm, and acrosome reaction. PCA revealed that sperm motility had a moderate to very robust correlation with other variables and is the most crucial parameter, accounting for 80.79% of all variables.

**Conclusion:**

The IGF-1 complex in Simmental bull seminal plasma was useful for increasing the fertility of post-thawed Kacang buck semen, and sperm motility was the principal component of semen fertility.

## Introduction

Kacang goat is a small ruminant, with a height of 55-65 cm. Adult male goats weigh 25-35 kg, whereas females weigh 20-30 kg. Morphologically, both male and female goats have horns, small and erect ears, and short neck [1]. They are white, black, brown, or a combination of those colors. They easily adapt to the weather and eat nuts or plants in farm environments. Puberty is reached at 8 months, the birth interval is 8.61 months, and the average litter size is 1.36 [2]. Kacang goats have been bred for generations in Indonesia and provide a wealth of local Indonesian livestock genetic resources that need to be protected and preserved [3].

Breeding Kacang goats using Artificial insemination (AI) is expected to accelerate population increases and preserve genetic diversity. AI enables the accelerated production of genetically valuable offspring and improves reproductive performance [4] for good human health and well-being. Unfortunately, frozen Kacang buck semen is unavailable yet as it is more susceptible to cold shock stress than that of other species [5]. Using the same extender and freezing protocol, Kacang buck sperm motility is more sensitive to cryo-damage than those of ram [6], Ettawa goat [7], and Simmental bull [8]. Without any antioxidant addition into the extender, approximately 60% of the post-thawed Kacang buck sperm ends with death [9]. Thereby, this level of post-thawed sperm motility has not met the requirement for AI use, which should be more than 40% [10].

Goat sperm is cryosensitive [5] due to the seminal plasma of goat semen itself [11]. In the freeze-thaw process, the lipase in the seminal plasma of goat semen reacts to phospholipids [12], weakening the interaction between the seminal plasma proteins and the sperm surface, decreasing seminal quality [13]. Meanwhile, bull seminal plasma contains the Insulin-Like Growth Factor-1 (IGF-1) complex. IGF-1 is a component of seminal plasma [14], secreted by Leydig and Sertoli cells [15]. Physiologically, seminal plasma proteins of bull semen protect post-ejaculated sperm in the epididymis and in does’ reproductive tract. IGF-1 is present in bovine seminal plasma, and IGF-1 receptors are expressed in spermatozoa, showing autocrine and paracrine activities at the cellular level [16]. Fluctuations in IGF-1 concentrations can affect the reproductive health of male animals through biological mechanisms [17]. IGF-1 forms 150-kDa ternary complexes [18], consisting of an IGF, IGF-binding protein, and acid-labile subunit [19]. Our earlier study has indicated that the crude protein of Simmental bull seminal plasma increased the quality of post-thawed semen of Kacang bucks [9]. Simmental bulls are a high fertility bull breed based on several bull fertility traits, such as concentration, the number of spermatozoa, motility, and the number of spermatozoa abnormalities [20].

To the best of our knowledge, the use of IGF-1 complexes from Simmental bull seminal plasma in skim milk-egg yolk (SM–EY) extender to enhance cryopreservation of Kacang buck semen has never been studied. Furthermore, this study was conducted to determine the effects of Simmental bull seminal plasma with IGF-1 complexes on the fertility of post-thawed semen of Kacang bucks based on sperm motility, viability, intact plasma membrane (IPM), MDA (malondialdehyde) levels, capacitation status, and acrosome reaction.

## Materials and Methods

### Ethical approval

This study was conducted based on the approval of the Animal Care and Use Committee, Airlangga University, Surabaya, Indonesia (no. 520/HRECC.FODM/VII/2019). The committee assessed this study’s proposal based on animal welfare principles, the Animals (Scientific Procedures) Act 1986 of the UK, EU Directive 2010/63/EU for animal experiments, and associated guidelines. The collection of bull and goat semen was conducted according to the protocol of Chapter 4.7 (the Collection and processing of bovine, small ruminant, and porcine semen) of the Terrestrial Animal Health Code of the World Organization for Animal Health.

### Study period and location

This study was conducted from October 2020 to February 2021 at the Regional AI Center (RAIC), a part of the Teaching Farm of the Faculty of Veterinary Medicine, Airlangga University. The RAIC is located in the village of Tanjung, a sub-district of Kedamean, Gresik District, East Java, Indonesia, at coordinates 7° 19′ 25′′ S and 112° 32′ 54′′ E. This village is a lowland area with an altitude of +11 m above sea level. The climate is wet tropical, with an average annual temperature of ±28.3°C and relative humidity of ±76%. The average rainfall per day is 31.76 mm, with an annual rainfall of 3017.2 mm, and there are 95-120 rainy days [21].

### Experimental animals

Two heads of Simmental bulls aged 5-6 years, weighing 400-900 kg, and four heads of Kacang buck aged 3-5 years, weighing 25-35 kg, were used for this study. The animals were reared at the Teaching Farm of the Faculty of Veterinary Medicine, Airlangga University, Surabaya. The semen of Simmental bulls was routinely collected for frozen semen production at the RAIC of Airlangga University.

### Semen collection

The semen of Simmental bulls and Kacang goats was collected twice a week using an artificial vagina. The fresh semen was examined macroscopically, assessing the following parameters: volume, pH, consistency, and color. In addition, it was examined microscopically, assessing the following parameters: Sperm viability, motility, and concentration. The semen that had at least 70% sperm motility and viability was used in the analysis [10,22]. A pool of eight Simmental bull ejaculates was used for identifying and isolating IGF-1 complex protein. Meanwhile, 12 Kacang buck ejaculates were subjected to this study. The ejaculate of each goat at every collection was divided into three parts of equal volume and diluted in an extender accordingly to the treatment groups.

### Purification of bull seminal plasma protein

Simmental bull ejaculates were dissolved in phosphate-buffered saline (PBS) 1:10 v/v and centrifuged at 363×g for 10 min at 5°C, and the supernatant was collected using a micropipette. Each 1-mL supernatant was added to 5-mL PBS and 5-mL phenylmethylsulfonyl fluoride, and the mixture was vortexed for 5 min, sonicated for 10 min at 4°C, vortexed again to homogenize it, and centrifuged at 4032×g for 10 min. The supernatant was added to absolute ethanol in the same volume and allowed to precipitate overnight. The ethanol was removed, and the pellet was added to Tris hydrochloride in the same volume [23].

### Identification and isolation of the IGF-1 complex

The IGF-1 complex was identified on purified seminal plasma protein using 12% Poly-Acrylamide Gel Electrophoresis (PAGE) with a broad-range sodium dodecyl sulfate–PAGE standard (6.5-212 kDa; BioLabs, New England) marker and stained using Coomassie Brilliant Blue R-250 [24]. Western blotting was performed using IGF-1 monoclonal antibody (Sigma-Aldrich, Missouri, USA) as the primary antibody [24], goat anti-bovine IGF-1 (Bio-Rad, California, USA) as a secondary anti-IGF-1 antibody [25], with chemiluminescence substrate and a C-Digit LI-COR [26] for detecting the IGF-1 complex protein. The IGF-1 complex protein was isolated using electro-elution at 130 V and 30 mA for 1 h. Spectrophotometric analysis (photometer; IMV Technologies, L’Aigle, France) was performed to measure the IGF-1 complex isolate [9,27]. The isolate was collected and stored at −70°C until further use.

### SM–EY extender

Ten grams of SM powder (115338; Merck Millipore, Massachusetts, USA) were dissolved in 100-mL distilled water, which was heated at 95°C for 10 min and then cooled to room temperature. Then, 5-mL homogenized EY (derived from laboratory chicken eggs), 1.000-IU/mL penicillin, and 1-mg/mL streptomycin were added to 95 mL of this solution [9]. The extender was divided equally into three groups: Without addition of the IGF-1 complex protein as the control group (T0); with the addition of 12-μg IGF-1 complex protein (T1); and with the addition of 24-μg IGF-1 complex protein per 100-mL extender (T2).

### Frozen semen

Each extender group was equally divided into two portions. The first portion was added to Kacang buck fresh semen to obtain 480 million spermatozoa/mL concentrations. The second portion was added to glycerol to obtain a concentration of 16% and was slowly added to the first portion at the same volume to obtain a concentration of 240 million spermatozoa/mL of extended semen. The extended semen was equilibrated at 5°C in a cold handling cabinet (minitube) for 1 h and was packaged in 0.25-mL French straws and sealed. The straws were exposed to liquid nitrogen vapor for 10 min and stored in liquid nitrogen for 2 weeks before they were assessed [9]**.**

### Semen fertility assessment

Twelve straws of frozen semen from each group (one straw was randomly taken as a representative of each ejaculate) were evaluated. The straws were thawed in sterile water at 37°C for 30 s to measure sperm motility, viability, IPM, MDA levels, capacitation status, and acrosome reaction. Sperm motility, viability, IPM, and MDA levels were assessed based on our earlier report [28].

### Sperm motility

A 10-μL semen sample was added to 1-mL physiological saline (0.9% sodium chloride solution w/v), homogenized, dropped on an object glass, and covered. The progressive motility of spermatozoa was observed under a light microscope (BX-53; Olympus Life Science, Tokyo, Japan), equipped with a computer-assisted sperm analyzer at 400× magnification [28].

### Sperm viability

One drop of 2% eosin was placed on the tip of the object glass, added with a drop of semen (10 μL), homogenized, and then smeared and fixed over the flame. The slide was examined at 400× magnification under a light microscope (BX-53; Olympus Life Science, Tokyo, Japan) on 100 sperm cells. Live sperm cells were characterized by their bright transparent heads. Meanwhile, the heads of dead sperm cells were reddish due to damage of the plasma membrane; thereby, the dye enters the head of the dead sperm [28].

### IPM

A 0.1-mL sample was diluted with a 1-mL hypoosmotic solution and incubated at 37°C for 30 min. The hypoosmotic solution was made of 1.352-g fructose and 0.735-g sodium citrate dihydrate dissolved in 100-mL distilled water. The IPMs of 100 sperm cells were counted per sample under a light microscope (BX-53; Olympus Life Science, Tokyo, Japan) at 400× magnification. The IPMs were indicated by circular tails, and damaged plasma membranes were indicated by straight tails [28].

### MDA levels

The Thiobarbituric acid (TBA) method was used for measuring MDA concentrations. The MDA kits contained 0, 1, 2, 3, 4, 5, 6, 7, and 8 μg/mL of TBA, and 100-μL semen samples were dissolved in distilled water to 550 μL, and 100-μL 20% trichloroacetic acid was added. Then, the mixture was homogenized for 30 s. A 250-μL 1 N HCl was added to the mixture and homogenized. A 100-μL 1% sodium thiobarbiturate was homogenized and then centrifuged at 31×g for 10 min. The supernatant was incubated in a water bath at 100°C for 30 min and then allowed to cool at room temperature. The absorbance of the kits and samples was measured at 533 nm using a spectrophotometer. The MDA concentration (ng/mL) was calculated based on the sample’s absorbance values extrapolated to the standard MDA curve [28].

### Capacitation and acrosome reaction

The sperm capacitation and acrosome reaction statuses were evaluated using chlortetracycline (CTC) fluorescence staining [29]. A 135-μL measure of the sample was added to 15-μL H33258 solution (10-μg H33258/mL PBS) and then incubated for 10 min at room temperature. A 250-μL 2% (w/v) polyvinylpyrrolidone (Sigma-Aldrich, Missouri, USA) in PBS was added and then centrifuged at 700×g for 5 min. The supernatant containing excess dye was discarded, and the pellet was resuspended in 100-μL PBS and 100-μL CTC solution (750-mM CTC in 5-μL buffer consisting of 20-mM Tris, 130-mM NaCl, and 5-mM cysteine at a pH of 7.4). The capacitation status was assessed using a fluorescence microscope (Nikon, Tokyo, Japan) for 100 spermatozoa per slide [30]. Incapacitated sperms were indicated by green fluorescence distributed uniformly over the entire sperm head, with or without a more robust fluorescent line at the equatorial segment. Meanwhile, capacitated sperms had green fluorescence over the acrosomal region and a dark post-acrosomal region. Acrosome-reacted sperms showed a mottled green fluorescence over the head, green fluorescence only in the post-acrosomal region, or no fluorescence over the head [29].

### Statistical analysis

The fertility of post-thawed semen was evaluated using analysis of variance, followed by Tukey’s honestly significant difference test at a 95% significance level in Statistical Package for the Social Sciences (version 23; IBM Corp., Armonk, NY, USA). The semen fertility assessed was the percentages of sperm motility, viability, IPM, MDA levels, incapacitated sperm, capacitated sperm, and sperm with acrosome reaction.

Principal component analysis (PCA) was conducted using the XLSTAT statistical software (version 2.9; Addinsoft, Paris, France). In PCA, the first analysis was the correlation between variables. Coefficient correlation (r) values were classified as follows: 0.00-0.19 is very weak; 0.20-0.39 is weak, 0.40-0.59 is moderate, 0.60-0.79 is robust, and 0.80-1.0 is very robust [31]. If the correlation value is more than 0.5, it can be concluded that multicollinearity or intercorrelation exists between variables. The second analysis is the Kaiser–Meyer–Olkin (KMO) measure of sampling, which compares the distance between the final correlation coefficient and the partial correlation coefficient. The KMO value is eligible if it is >0.5 [32]. Furthermore, correlation matrix analysis (correlation between variables) was performed using Bartlett’s test of sphericity and Measure of sampling adequacy (MSA). Barlett’s test of specificity value must meet the significance requirement of <0.05. Meanwhile, a variable with an MSA value of >0.5 is valid and can be used for further analysis [33].

Eigenvalue analysis was performed to determine which variables were dominant among all variables. Variables that have eigenvalues of >1 represent all variables. The cumulative value of the resulting proportion shows the percentage of these variables representing all other variables. The distribution of eigenvalues is described in the form of a scree plot. After determining the principal factor, then to determine which variable will enter into which principal factor, factor rotation analysis was conducted in the form of a PCA biplot. In factor rotation analysis, the variables are communal in a principal factor if the average communality value is >0.5, indicating that all variables can explain the factor. The characteristics of variable distribution based on factor analysis results are shown using factor rotation diagrams (biplots). In PCA, the newly formed factor will become a new component or variable used for other analysis purposes [34].

## Results

**Figure-1:**
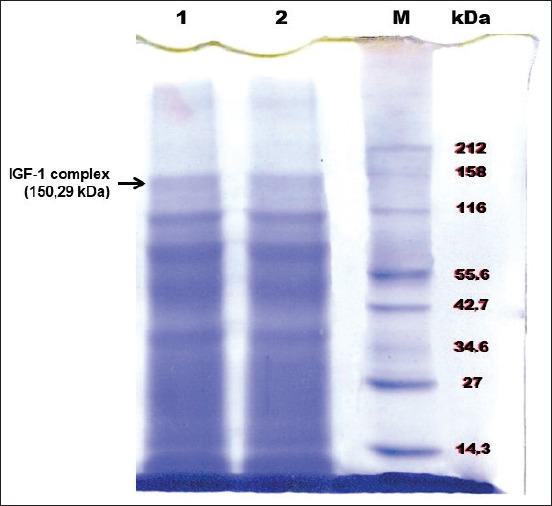
Sodium dodecyl sulfate-polyacrylamide gel electrophoresis (SDS-PAGE) of Simmental bull seminal plasma protein. kDa: Kilo Dalton, M: Marker, 1 and 2: Seminal plasma of first and second Simmental bulls.

**Figure-2:**
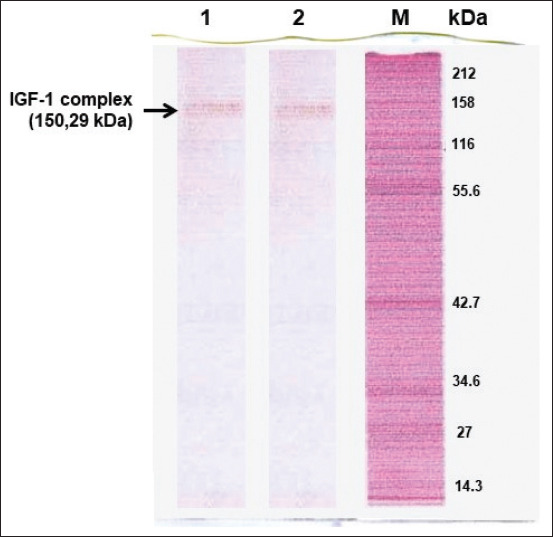
Western blot of IGF-1 complex protein of Simmental bull seminal plasma protein. kDa: Kilo Dalton, M: Marker, 1 and 2: Seminal plasma of first and second Simmental bulls.

Five protein bands of Simmental bull seminal plasma were found (Figure-1). The 150.29-kDa band was verified as an IGF-I complex on Western blotting using an IGF-1 monoclonal antibody (Figure-2).

The ejaculate volume, concentration, viability, motility, IPM, and morphologic abnormality of the fresh semen of Kacang buck were examined (Table-1). The freeze-thaw process causes a dramatic decline in semen fertility from fresh ejaculates (Table-1) compared with post-thawed semen extended in SM–EY without the addition of an IGF-1 complex. A decline of 72.02% progressive motility (from 91.45% to 25.59%), 69.50% sperm viability (from 93.25% to 28.44%) (T0 in Table-2), and 68.13% IPM (from 84.3% to 26.87%) (T0 in Table-3) was observed. The addition of the IGF-1 complex protein to the SM–EY extender inhibited those declines.

**Table-1:** The characteristics of Kacang buck fresh semen.

Indicator	Average
Volume (mL)	1.9±0.15
Concentration (juta) sel spz/mL	3975±255
Sperm viabilities (%)	93.25±0.15
Sperm motility (%)	91.45±0.35
Intact plasma membrane (%)	84.30±0.50
Morphologic abnormalities (%)	3.00±0.05

**Table-2:** The sperm motility and viability of post-thawed Kacang buck semen in skim milk-egg yolk extender with and without the addition of IGF-1 complex protein.

Group	Motility	Viability
T0	25.59±1.35^c^	28.44±1.64^c^
T1	43.73±1.93^a^	44.43±1.40^a^
T2	31.77±1.40^b^	33.96±1.46^b^

Different superscript letters in a column indicate significant differences (p<0.05), T0=Skim milk-egg yolk extender without the IGF-1 complex protein; T1=Skim milk-egg yolk extender with 12 µg of IGF-1 complex protein/100 mL extender; T2=Skim milk-egg yolk extender with 24 µg of IGF-1 complex protein/100 mL extender.

**Table-3:** The sperm intact plasma membrane (IPM) and malondialdehyde (MDA) levels of post-thawed Kacang buck semen in skim milk-egg yolk extender with and without the addition of IGF-1 complex protein.

Group	IPM	MDA
T0	26.87±1.57^c^	2698.80±25.95^a^
T1	41.56±2.21^a^	1911.93±51.34^c^
T2	32.50±2.17^b^	2498.75±106.87^b^

Different superscript letters in a column indicate significant differences (p<0.05), T0=Skim milk-egg yolk extender without the IGF-1 complex protein; T1=Skim milk-egg yolk extender with 12 µg of IGF-1 complex protein/100 mL of the extender; T2=Skim milk-egg yolk extender with 24 µg of IGF-1 complex protein/100 mL of extender.

**Figure-3:**
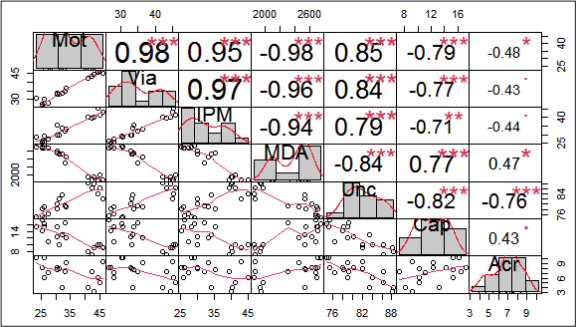
Intercorrelation among variables. Mot; sperm motility, Via: Sperm viability, IPM: Intact plasma membrane, MDA: Malondialdehyde, Unc: Uncapacitated sperm, Cap: Capacitated sperm, Acr: Sperm with acrosome reaction. ***p<0.001, **p<0.01, *p<0.05.

Correlation analysis (Figure-3) showed that among the 21 variables, 16 (76.19%) had a correlation value of more than 0.5 (range, r=0.71-0.98), and only five (23.81%) had a correlation value of lower than 0.5 (range, r=0.43-0.48). Sperm motility had a very robust positive correlation (r>0.80) with sperm viability and IPM and a very robust negative correlation with incapacitated sperm and MDA levels (p<0.001). Capacitated sperm had a robust positive correlation (r=0.60-0.79) (p<0.001), whereas sperm with acrosome reaction had a moderate positive correlation (r=0.40-0.59) (p<0.05) with sperm motility.

**Figure-4:**
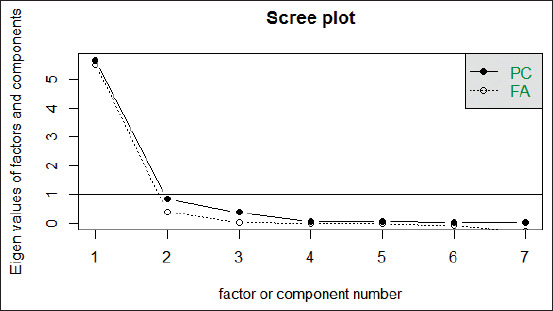
Scree plot displayed factor or component based on Eigenvalues. Factor 1: Sperm motility, 2: Sperm viability, 3: Intact plasma membrane, 4: Malondialdehyde, 5: uncapacitated sperm, 6: Capacitated sperm, 7: Sperm with acrosome reaction. PC: Principle factor, FA: A factor analysis.

The KMO measure of sampling was 0.8, and the MSA values ranged from 0.53 to 0.94. It means that all variables were valid and can be used for further analysis. Barlett’s test of sphericity obtained a value of 185.89 with a significance of <2.2×10-16 (p<0.05). The scree plot showed that only one variable (sperm motility) reached an eigenvalue of higher than 1 (Figure-4), with a cumulative value of the proportion of 80.79%. It means that sperm motility represents 80.79% of all variables.

**Figure-5:**
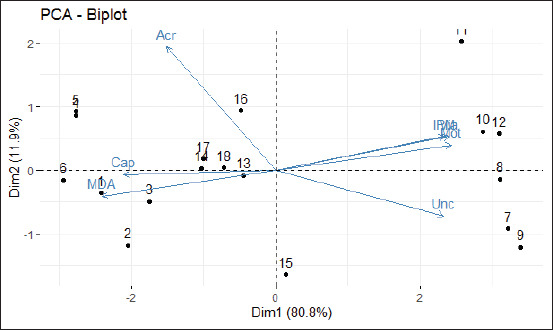
PCA-Biplot shows the characteristics of the distribution of variables based on the results of factor analysis. Dim1: Dimension 1 with the cumulative proportion of PC1 (sperm motility). Dim2: Dimension 2 with a proportion of variance PC2 (sperm viability). Mot: Sperm motility, Via: Sperm viability, IPM: Intact plasma membrane, MDA: Malondialdehyde, Unc: Uncapacitated sperm, Cap: Capacitated sperm, Acr: Sperm with acrosome reaction. The characteristics of the distribution of variables based on factor analysis results (Figure-5) show that sperm motility can represent all variables of 80.8% as a sperm fertility variable.

The average communality value of all variables was 0.81 (range, 0.37-0.95), which means that all variables were communal in the percentage of sperm motility. Only one variable (sperm motility) was determined as a principal variable of semen fertility, and thereby, rotation factor analysis was not needed. However, the PCA biplot was presented as a visualization of the distribution characteristics of the variables (Figure-5). The PCA biplot showed dimension 1 with a cumulative proportion of PC1 (sperm motility) of 80.79% as ordinate and dimension 2 with a proportion of variance PC2 (sperm viability) of 11.87% as abscissa. The distribution of the variables as vectors in quadrants revealed the value of correlation coefficient among them.

### Sperm motility and viability

The addition of IGF-1 in SM–EY has resulted in higher (p<0.05) post-thawed sperm motility and viability of Kacang buck semen than that SM–EY without IGF-1 only (T0 group). The addition of 12-μg IGF-1 complex protein/100-mL SM–EY extender (T1) resulted in the highest post-thawed motility and viability. A higher dose of the IGF-1 complex protein (T2 group; 24-μg IGF-1 complex protein/100-mL SM–EY extender) showed higher post-thawed sperm motility and viability of Kacang buck semen than those of the T0 group. However, it was lower than that of the T1 group (Table-2).

### IPM and MDA levels

The post-thawed sperm IPM and MDA levels of Kacang buck semen extended in SM–EY only (T0 group) had the lowest fertility. The addition of 12-mg IGF-1 complex protein/100-mL SM–EY extender resulted in the highest post-thawed IPM and the lowest MDA levels. A higher dose of the IGF-1 complex protein (T2 group; 24-μg IGF-1 complex protein/100-mL SM–EY extender) showed a higher post-thawed IPM and the lowest MDA levels. However, in the T2 group, the IPM was lower, and the MDA levels were higher than those in the T1 group (Table-3).

### Sperm capacitation status and acrosome reaction

The addition of 12-μg IGF-1 complex protein/100-mL SM–EY extender resulted in the highest proportion of incapacitated post-thawed sperm compared with the other groups. A higher dose of IGF-1 (T2 group) showed a higher proportion of incapacitated post-thawed sperm than semen extended in SM–EY only (T0 group). However, in the T2 group, there were fewer incapacitated sperm and more capacitated sperm than in the T1 group. The post-thawed sperm acrosome reactions of the T1 and T2 groups were not significantly different (p>0.05), and both groups showed more sperm acrosome reactions than the T0 group (Table-4).

**Table-4:** The capacitation status and acrosome reaction of sperm of post-thawed Kacang buck semen in skim milk-egg yolk extender with and without the addition of the IGF-1 complex protein.

Group	Uncapacitated	Capacitated	Acrosome reaction
T0	76.48±1.71^c^	14.91±2.15^a^	8.36±1.63^a^
T1	85.91±2.39^a^	8.99±1.20^c^	5.86±2.25^b^
T2	80.42±2.02^b^	13.29±2.26^b^	7.43±1.75^b^

Different superscript letters in a column indicate significant differences (p<0.05), T0=Skim milk-egg yolk extender without the IGF-1 complex protein; T1=skim milk-egg yolk extender with 12 µg of IGF-1 complex protein/100 mL extender; T2=skim milk-egg yolk extender with 24 µg of IGF-1 complex protein/100 mL of extender

## Discussion

Bulls with high fertility demonstrate a high antioxidant capacity of seminal plasma protein [35,36]. The 150.29-kDa band of the Simmental bull seminal plasma (Figure-1) was verified as an IGF-I complex on Western blotting using IGF-1 monoclonal antibody (Figure-2). Simmental bulls (from which the IGF-1 complex originated) were categorized as elite bulls based on our earlier study [8]. The frozen semen of Simmental bulls used in this study is preferred among inseminators in several areas of Indonesia.

Bovine seminal plasma proteins are secreted by the seminal vesicles and are associated with the fertility of bulls [37]. Physiologically, seminal plasma acts as a medium to spermatozoa, providing energy for metabolism and motility, buffering against pH changes, regulating and controlling capacitation [14]**,** establishing sperm reservoirs, and providing protection from a female’s immune system [38]. Seminal plasma proteins are related to major functions involved in sperm cell protection, sperm motility, capacitation, and acrosome reaction [39]. Among several proteins in the seminal plasma, IGF-1 is a polypeptide of 70 amino acids secreted by Leydig and Sertoli cells [40]. IGF-1 is a potent mitogenic, anabolic substance [41], and regulator of male infertility [42]. Most IGFs are not composed of free molecules, but they are formed by a complex of IGF-binding protein and acid-labile subunit [18,43] as a 150-kDa ternary complex [44], which has a longer half-life than free IGF-1 [19]. Circulating the biological activity of IGFs is an autocrine or paracrine process at the cellular level [16]. It may be executed independently [19] for the survival of sperm [45] and to increase the duration of sperm motility of fresh semen [46]. IGF-1 in the seminal plasma improves sperm motility by reducing oxidative stress, maintaining structural membrane integrity and mitochondrial membrane potential, and protecting calmodulin, dermcidin, and the sperm acrosome membrane [47].

PCA statistics have been used to determine the seminal traits affecting bull fertility [48], the differentiating factors of bull fertility [49], the role of seminal plasma proteins in sperm motility of buffalo [50], morphometric analysis of sperm by different separation methods [51], changes in bull semen metabolome concerning cryopreservation and fertility [52], the cellular and functional parameters on the freezability of sperm [53], proteomic and lipidomic tolerance of stored boar spermatozoa to autologous seminal plasma [54], and spermatozoa quantitative proteomics of human normozoospermia and asthenozoospermia [55]. To the best of our knowledge, using PCA to determine the fertility of post-thawed extended semen has never been studied.

Sperm motility was the only principal component of sperm fertility, representing 80.79% of all variables (Figure-4). The other variables have a moderate to very robust correlation with sperm motility. Sperm viability, IPM, and incapacitated sperm had a very robust positive correlation with sperm motility. Meanwhile, MDA levels had a very robust negative correlation, capacitated sperm had a robust negative correlation, and sperm with the acrosome reaction had a moderate correlation with sperm motility (Figures-3 and 5). The average sperm motility and viability of Kacang buck fresh semen were more than 70% (Table-1). Therefore, it qualified for freezing for AI use [10]**.** Seminal plasma of buck semen contained proteins involved in metabolic pathways of energy production for sperm motility, acrosome reaction, sperm capacitation, and reduction of oxidative stress [56]. However, the freeze-thaw process causes a dramatic decline in semen fertility in fresh ejaculate compared with post-thawed semen extended in SM–EY without the addition of IGF-1. In addition, these proteins could not prevent cell damage to Kacang buck semen due to the freeze-thaw process. The results of our earlier study have shown that the viability of post-thawed Kacang buck semen without any antioxidant [9] is lower than that of Ettawa goat [7], ram [28], and Simmental bull semen [8]. The sperm freezing process results in changes in the ultrastructure, biochemistry, and function of spermatozoa. Damaged spermatozoa membranes increase sperm membrane permeability, hyperoxidation, and Reactive Oxygen Species (ROS) formation [57]. The significant decrease in antioxidant content indicates antioxidative system disruption during the freeze-thaw process [56]. The addition of antioxidants improves the quality of post-thawed semen [58].

The cryopreservation of semen first affects the polyunsaturated fatty acid of the sperm plasma membrane, characterized by the formation of lipid peroxidation, which disrupts the sperm plasma membrane [59]. The primary products of lipid peroxidation are lipid hydroperoxides, and MDA is a secondary product. MDA is an end-product generated by the decomposition of arachidonic acid and more prominent polyunsaturated fatty acids. Therefore, MDA is a biomarker for lipid peroxidation [60], which explains why the change in the IPM was contrary to that of MDA levels. PCA revealed that IPM was negatively strongly correlated (r=−0.94) with MDA levels. The post-thawed IPM and MDA levels of Kacang buck semen extended in SM–EY only (T0 group) were lower than those of the other groups (Table-3).

The addition of 12-μg IGF-1 complex protein/100-mL SM–EY extender (T1) resulted in the highest post-thawed IPM and the lowest MDA levels (Table-3). The IGF-1 in the extender binds to IGF-1 receptors in the surface of the sperm membrane [61] to maintain the structural integrity of post-thawed plasma membrane semen [17]. The intactness of the plasma membrane is essential for sperm viability and sperm motility [62]. Moreover, the lowest post-thawed IPM was followed by the highest MDA levels and the lowest sperm viability and sperm motility (T0). However, the highest plasma membrane intactness was followed by the lowest MDA levels and the highest sperm viability and sperm motility (Table-3).

A higher dose of the IGF-1 complex protein (T2 group) showed a higher IPM and the lowest MDA levels in the post-thawed sperm. However, in the T2 group, the IPM was lower, and the MDA levels were higher than those of the T1 group (Table-3), which was due to the antioxidant paradox [63,64], as explained earlier.

The freeze-thaw process causes damage to the sperm plasma membrane first, which is characterized by low IPM and high levels of MDA. It was the initial cause of impaired semen fertility, followed by a decrease in sperm motility and viability. However, based on PCA, it was not IPM and MDA, but sperm motility was the principal variable of buck semen fertility (Figure-4). IPM had a very robust positive correlation (r=0.95), and MDA levels had a very robust negative correlation (r=−0.98) with sperm motility (Figures-3 and 5). This result conforms to those of an earlier study where motility was an important parameter of male fertility [65]. The quality standard for post-thawed goat semen is a minimum of 40% sperm motility in 50 million sperm per dose for AI, according to the Indonesian National Standard Agency [10]. Without the IGF-1 complex protein (T0), the motility of post-thawed Kacang buck semen in SM–EY extender was 25.59±1.35% (Table-2), which did not qualify for AI. The IGF-1 complex protein in SM–EY resulted in higher post-thawed sperm viability and motility of Kacang buck semen than those in the T0 group. This finding conforms to the results of a previous study where IGF-1 improved sperm motility and viability of bovine frozen-thawed semen. Post-thawed progressive motility was higher in boars when IGF-1 was added, but no effect was observed on boar sperm viability [66]. The IGF-1 complex protein binds to receptors on the sperm plasma membrane [45], increasing sperm motility by increasing mitochondrial membrane potential [61]. However, IGF-1-protected sperm in cryopreservation was not due to direct antioxidant activity [67], but indirectly through the increase in calcium ions in the cells [44]. Calcium ions regulate ROS homeostasis, activate antioxidant enzymes, increase the level of SOD, and induce mitochondrial glutathione release. Meanwhile, calmodulin (a calcium-binding protein) interacts with antioxidant enzymes involved in ROS homeostasis [68].

Antioxidants lower the acid phosphatase enzyme and decrease cellular death or damage [69]. Antioxidants also affect cholesterol efflux and tyrosine phosphorylation, improving sperm motility [70]. Sperm motility requires energy produced by the mitochondria through energy metabolism by increasing glucose uptake and pyruvate dehydrogenase activity, and it has an antioxidant effect when stored [71]. IGF-1 promotes glucose uptake as a substrate to produce energy in the mitochondria [72] and indirectly contributes to antioxidant action. In addition, calcium ions, together with cAMP (cyclic Adenosine Monophosphate) due to indirect actions of IGF-1, regulates flagella motions [73]. This finding conforms to the results of an earlier study where the progressive motility of post-thawed stallion semen is higher when IGF-I is added to the extender [74].

The addition of 12-μg IGF-1 complex protein/100-mL SM–EY extender resulted in the highest post-thawed sperm motility and viability. PCA revealed a robust correlation (r=0.98) between sperm motility and viability (Figures-3 and 5). Spermatozoa motility was higher in sperm samples with a high concentration of seminal IGF-1, and higher concentrations of IGF-1 are associated with higher sperm motility [45]. In addition, the concentration of the IGF-1 complex protein in seminal plasma is positively correlated with the concentration, morphology, and motility of sperm [75]. However, in this study, a higher dose of IGF-1 (T2 group) showed higher motility and viability of Kacang buck semen than the T0 group, but sperm motility and viability of the T2 group were lower than those of the T1 group sperm (Table-2). This may be attributed to higher exposure to antioxidants, resulting in an antioxidant paradox [63], which causes a lack of ROS for the physiological functioning of sperm [64].

PCA revealed that the percentage of incapacitated sperm had a very robust negative correlation (r=−0.82) with the percentage of capacitated sperm and a robust negative correlation (r=−0.76) with the percentage of sperm with acrosome reaction (Figures-3 and 5). The spermatozoa must experience capacitation to bind, penetrate, and fertilize the egg. Capacitation is triggered by sterol-binding albumin, lipoproteins, and proteolytic and glycosidase enzymes, such as heparin, secreted by endometrial cells. These secretions activate sperm-specific calcium ion channels, resulting in calcium influx, stimulating a cascade reaction for capacitation [76]. The increase in calcium ion permeability is followed by the increase in the strength and speed of sperm flagellum moving for a swim [29]. Faster spermatozoa have higher energy needs derived from cAMP-dependent tyrosine phosphorylation in the mitochondria, producing ROS [77]. Low ROS levels also trigger and regulate a series of events, including protein phosphorylation for capacitation [78]. Unfortunately, cryopreservation causes a loss of viability to approximately half of the sperm population, and the remaining motile sperm in a state of premature capacitation reduces the binding ability [76]. The seminal plasma of buck semen shows more significant protein abundance that inhibits premature sperm capacitation [56]. However, cryopreservation inactivates these proteins [79].

The addition of 12-μg IGF-1 complex protein/100-mL of SM–EY extender (T1) resulted in the highest proportion of incapacitated post-thawed sperm and the lowest proportion of capacitated sperm compared with other groups (Table-4). Antioxidants release an electron to rampaging free radicals to prevent oxidative damage [80]. IGF-1 may be involved in the antioxidant protection pathways of sperm cells [81]. Unraveling the genomic architecture found that the association analyses identified IGF-1 receptors, with functions related to calcium channel regulation in sperm [61]. The IGF-1 complex binds to the IGF-1 receptor on the sperm membrane [45]. However, the effects of adding IGF-1 to sperm in the freeze-thawed process are not due to the direct antioxidant activity [67]. Indirect IGF-1 antioxidant activity may be related to the effects of IGF-1 to increase calcium ions in the cells [44], which is contrary to the process of capacitation. A positive correlation was found between circulating IGF-1 complex and serum calcium [82]. A higher dose of the IGF-1 complex protein in the T2 group showed lower proportions of incapacitated post-thawed sperm and higher proportions of capacitated sperm than the T1 group (Table-4). This finding can be explained by the antioxidant paradox [64].

Capacitation is an essential physiological prerequisite for sperm cell acrosome reaction and oocyte fertilization. During capacitation, the plasma membrane’s destabilization prepares the sperm’s tip for acrosome reaction [77]. The cryo-capacitated sperm plasma membrane is fragile and unresistant to spontaneous acrosome reaction and deterioration and fails to fertilize an ovum [83].

Acrosome integrity is vital in preventing premature loss of acrosomal enzymes and maintaining the fertilization capacity of the sperm [84]. When sperm reaches the ovum’s zona pellucida, the actin filament of the sperm will bind to the zona pellucid-3 of the ovum, followed by the release of hydrolytic enzymes from the sperm’s acrosome. The enzymes used to digest the zona pellucida allow the male’s material genetic entrance into the ovum’s cytoplasm [30]. Sperm needs a low ROS level to preserve the acrosome reaction [85]. These oxygen free radicals have crucial stimulatory effects on the cAMP/PKA/tyrosine phosphorylation cascade and the preparation of sperm to undergo acrosomal exocytosis [79]. However, cryopreservation causes lipid peroxidation, higher production of ROS and MDA, and damage to the plasma membrane, acrosome [62], and phosphorylated proteins [86], and disrupts some membrane functions [87]. Excessive ROS cause calcium overload, mitochondrial depolarization, cytochrome C release, lipid peroxidation, transcription factor activation, and DNA damage, and leading to apoptotic and non-apoptotic cell death [88]. Cryopreservation induces precocious capacitation, followed by premature acrosome reaction [83], which causes sperm to lose viability before attachment to the zona pellucida of an ovum [76]. In the control group (T0), the percentage of the premature acrosome reaction in post-thawed sperm was higher than in other groups. The addition of 12- or 24-μg IGF-1 complex protein/100-mL SM–EY extender resulted in a lower premature acrosome reaction (Table-4).

PCA revealed that percentages of incapacitated sperm, capacitated sperm, and sperm with the acrosome reaction have a very robust positive (r=0.85), robust negative (r=−0.79), and moderate negative (r=−0.49) correlations with the percentage of sperm motility, respectively (Figures-3 and 5). Incapacitated post-thawed sperm changes among groups were similar to those of sperm viability, motility, and IPM. Meanwhile, post-thawed capacitated sperm and acrosome reaction changes among the groups were linearly associated with those of MDA levels.

## Conclusion

To the best of our knowledge, this was the first study on the effects of the addition of IGF-1 derived from Simmental bull seminal plasma extended in SM–EY on the fertility of post-thawed Kacang buck semen and analyzed using PCA statistics. Sperm motility was the only principal variable of Kacang buck semen fertility, representing 80.79% of all variables (including sperm viability, IPM, MDA levels, capacitation status, and acrosome reaction of sperm). The overall conclusion was that IGF-1 from Simmental bull seminal plasma is useful in increasing the fertility of post-thawed Kacang buck semen.

## Authors’ Contributions

SS: Compiled ideas and designed this text’s main framework. SS: Research work under IM’s supervision. SS and INT: Processed and evaluated the post-thawed semen fertility (viability, motility, IPM, WW, SU, and RR: Processed the measurement of MDA levels, staining, and assessment for sperm acrosome reaction and capacitation. SS and IM: Conducted the statistical analysis and conceived the manuscript. WW, SU, and RR: Critically read and revised the manuscript for intellectual content. All authors read and approved the final manuscript.

## References

[1] Azmidaryanti R, Misrianti R, Siregar S (2017). Comparison morphometric of kacang goat in intensive and semi intensive system in Kampar, Riau province. J. Anim. Prod. Sci. Technol.

[2] Wati L, Aka R, Saili T (2014). Kid crop of Kacang Goat in North Konawe Regency, Southeast Sulawesi province, Indonesia. J. Trop. Anim. Sci. Technol.

[3] Suswono S (2012). Decree of the Minister of Agriculture of the Republic of Indonesia. http://www.bibit.ditjenpkh.pertanian.go.id/sites/default/files/Kambing%20Kacang.pdf.

[4] Agossou D.J, Koluman N (2018). The effects of natural mating and artificial insemination using cryopreserved buck semen on reproductive performance in Alpine goats. Arch. Anim. Breed..

[5] Lv C, Wu G, Hong Q, Quan G (2019). Spermatozoa cryopreservation:State of art and future in small ruminants. Biopreserv. Biobank..

[6] Srianto P, Dahnia N, Samik A, Setyono H (2011). Motility, viability and plasma membrane intactness of post thawing Thick Tail Sheep in three variance diluter. Vet. Med.

[7] Suprayogi T.W, Susilowati S (2018). The effect of cattle seminal plasma crude protein on the cryopreservation of goat semen. Iran. J. Appl. Anim. Sci.

[8] Susilowati S, Sardjito T, Mustofa I, Widodo O.S, Kurnijasanti R (2021). Effect of green tea extract in extender of Simmental bull semen on pregnancy rate of recipients. Anim. Biosci..

[9] Susilowati S, Triana I.N, Sardjito T, Suprayogi T.W, Wurlina W, Mustofa I (2020). Effect of Simmental bull seminal plasma protein in egg yolk-citrate extender on Kacang buck semen fertility. Cryobiology.

[10] Indonesian National Standard Agency (2014). Frozen Semen-Part 3:Goat and Sheep. http://www.bibit.ditjenpkh.pertanian.go.id/sites.

[11] Kaewkesa T, Sathanawongs A, Oranratnachai A, Sumretprasong J (2016). The goat semen quality after being frozen using albumin and cholesterol substituted for egg yolk in semen extender. Thai. J. Vet. Med.

[12] Silva R.A, Batista A.M, Arruda L.C, Souza H.M, Nery I.H, Gomes W.A, Soares P.D, Silva S.V, Guerra M.M (2019). Concentration of soybean lecithin affects short-term storage success of goat semen related with seminal plasma removal. Anim. Reprod.

[13] Ramírez-Vasquez R.R, Cano A, Hozbor F.A, Cesari A (2019). Cryopreservation and egg yolk extender components modify the interaction between seminal plasma proteins and the sperm surface. Theriogenology.

[14] Velho A.L, Menezes E, Dinh T, Kaya A, Topper E, Moura A. A, Memili E (2018). Metabolomic markers of fertility in bull seminal plasma. PLoS One.

[15] Mora Rodríguez J.A, Porchia L.M, Camargo F, López-Bayghen E (2019). The use of insulin-like growth factor 1 improved the parameters of the seminogram in a patient with severe oligoasthenoteratozoospermia. SAGE Open Med. Case Rep.

[16] Cai Q, Dozmorov M, Oh Y (2020). IGFBP-3/IGFBP-3 receptor system as an anti-tumor and anti-metastatic signaling in cancer. Cells.

[17] Van Tran L, Malla B.A, Kumar S, Tyagi A.K (2017). Polyunsaturated fatty acids in male ruminant reproduction-a review. Asian Aust. J. Anim. Sci.

[18] Ding H, Wu T (2018). Insulin-like growth factor binding proteins in autoimmune diseases. Front. Endocrinol..

[19] Allard J.B, Duan C (2018). IGF-binding proteins:Why do they exist and why are there so many?. Front. Endocrinol..

[20] Butler M.L, Bormann J.M, Weaber R.L, Grieger D.M, Rolf M.M (2019). Selection for bull fertility:A review. Transl. Anim. Sci.

[21] Dinas Pekerjaan Umum dan Tata Ruang Kabupaten Gresik (2018). Infrastructure Development Plan Medium Term Gresik district, 2019-2023. The Government of Gresik District. https://www.sippa.ciptakarya.pu.go.id/sippa_online/ws_file/dokumen/rpi2jm/DOCRPIJM_1540439279002__Bab_2_Profil_Kabupaten_Gresik.pdf.

[22] Indonesian National Standard Agency (2017). Frozen Semen-Part 1:Bovine Bull. Indonesian National Standardized Agency, Jakarta, Indonesia. https://www.bibit.ditjenpkh.pertanian.go.id/sites/default/files/SNI%204869-1-2017.

[23] Aulanni'am A (2005). Proteins and Its Analysis.

[24] Mishra M, Tiwari S, Gomes A.V (2017). Protein purification and analysis:Next generation western blotting techniques. Expert Rev. Proteomics.

[25] Cima-Cabal M.D, Vazquez F, de Los Toyos J.R, Del Mar García-Suárez M (2019). Protein expression analysis by western blot and protein-protein interactions. Methods Mol. Biol.

[26] Bass J.J, Wilkinson D.J, Rankin D, Phillips B.E, Szewczyk N.J, Smith K, Atherton P.J (2017). An overview of technical considerations for Western blotting applications to physiological research. Scand. J. Med. Sci. Sports.

[27] Avan A.N, Demirci Çekiç S, Uzunboy S, Apak R (2016). Spectrophotometric determination of phenolic antioxidants in the presence of thiols and proteins. Int. J. Mol. Sci.

[28] Susilowati S, Wurlina W, Anom Adnyana I.D.P, Mustofa I, Hariadi M (2019). Physiological study of the use of seminal bull plasma in skim milk diluent to improve quality of frozen ram semen. Eur. J. Biosci.

[29] Ded L, Dostalova P, Zatecka E, Dorosh A, Komrskova K, Peknicova J (2019). Fluorescent analysis of boar sperm capacitation process *in vitro*. Reprod. Biol. Endocrinol..

[30] Kwon W.S, Shin D.H, Ryu D.Y, Khatun A, Rahman M.S, Pang M.G (2018). Applications of capacitation status for litter size enhancement in various pig breeds. Asian Aust. J. Anim. Sci..

[31] Rouaud M (2017). Probability, Statistics, and Estimation. Creative Commons Attribution-Non Commercial 4.0 International License (CC BY-NC 4.0).

[32] Banda T.D, Kumarasamy M (2020). Application of multivariate statistical analysis in the development of a surrogate water quality index (WQI) for South African Watersheds. Water.

[33] Rahman M.A.T, Hoque S, Saadat A.H.M (2017). Selection of minimum indicators of hydrologic alteration of the Gorai river, Bangladesh using principal component analysis. Sustain. Water Resour. Manage.

[34] Jolliffe I.T, Cadima J (2016). Principal component analysis:A review and recent developments. Philos Trans. A Mathe. Phys. Eng. Sci..

[35] Vickram A.S, Rajeswari V.D, Pathy M.R, Sridharan T.B (2016). Analysis of seminal plasma proteins of South Indian Jersey and Hybrid Bulls and their correlation with semen quality. Asian J. Anim. Sci.

[36] Nongbua T, Guo Y, Edman A, Humblot P, Morrell J.M (2018). Effect of bovine seminal plasma on bovine endometrial epithelial cells in culture. Reprod. Domest. Anim.

[37] Pardede B.P, Agil M, Supriatna I (2020). Protamine and other proteins in sperm and seminal plasma as molecular markers of bull fertility. Vet. World.

[38] Druart X, Rickard J.P, Tsikis G, de Graaf S.P (2019). Seminal plasma proteins as markers of sperm fertility. Theriogenology.

[39] Viana A, Martins A, Pontes A.H, Fontes W, Castro M.S, Ricart C, Sousa M.V, Kaya A, Topper E, Memili E, Moura A.A (2018). Proteomic landscape of seminal plasma associated with dairy bull fertility. Sci. Rep..

[40] Lee H.S, Park Y.S, Lee J.S, Seo J.T (2016). Serum and seminal plasma insulin-like growth factor-1 in male infertility. Clin. Exp. Reprod. Med..

[41] Simopoulou M, Philippou A, Maziotis E, Sfakianoudis K, Nitsos N, Bakas P, Tenta R, Zevolis E, Pantos K, Koutsilieris M (2018). Association between male infertility and seminal plasma levels of growth hormone and insulin-like growth factor-1. Andrologia.

[42] Lewitt M.S, Boyd G.W (2019). The role of insulin-like growth factors and insulin-like growth factor-binding proteins in the nervous system. Biochem. Insights.

[43] Haywood N.J, Slater T.A, Matthews C.J, Wheatcroft S.B (2019). The insulin-like growth factor and binding protein family:Novel therapeutic targets in obesity and diabetes. Mol. Metab..

[44] Beigi Harchegani A, Irandoost A, Mirnamniha M, Rahmani H, Tahmasbpour E, Shahriary A (2019). Possible mechanisms for the effects of calcium deficiency on male infertility. Int. J. Fertil. Steril..

[45] Ipsa E, Cruzat V.F, Kagize J.N, Yovich J.L, Keane K.N (2019). Growth hormone and insulin-like growth factor action in reproductive tissues. Front. Endocrinol..

[46] Zangeronimo M.G, Silva D.M, Murgas L.D.S, Sousa R.V, Rocha L.G.P, Pereira B.A, Faria B.G, Veras G.C (2013). Identification of insulin-like growth factor-I in boar seminal plasma and its influence on sperm quality. Arch. Zootec.

[47] Selvaraju S, Krishnan B.B, Archana S.S, Ravindra J.P (2016). IGF1 stabilizes sperm membrane proteins to reduce cryoinjury and maintain post-thaw sperm motility in buffalo (*Bubalus bubalis*) spermatozoa. Cryobiology.

[48] Panda S.K, Nayak G, Mishra C (2020). Meta-analysis of seminal traits affecting bull fertility. Trop. Anim. Health Prod.

[49] Kumaresan A, Johannisson A, Al-Essawe E.M, Morrell J.M (2017). Sperm viability, reactive oxygen species, and DNA fragmentation index combined can discriminate between above-and below-average fertility bulls. J. Dairy Sci.

[50] Codognoto V.M, Yamada P.H, Schmith R.A, de Ruediger F.R, Scott C, de Faria Lainetti P, Brochine S, de Paula Freitas-Dell'Aqua C, de Souza F.F, Oba E (2018). Functional insights into the role of seminal plasma proteins on sperm motility of buffalo. Anim. Reprod. Sci.

[51] Rubessa M, Kandel M.E, Schreiber S, Meyers S, Beck D.H, Popescu G, Wheeler M.B (2020). Morphometric analysis of sperm used for IVP by three different separation methods with spatial light interference microscopy. S*yst Biol. Reprod. Med*.

[52] Longobardi V, Kosior M.A, Pagano N, Fatone G, Staropoli A, Vassetti A, Vinale F, Campanile G, Gasparrini B (2020). Changes in bull semen metabolome in relation to cryopreservation and fertility. Animals.

[53] Hitit M, Ugur M.R, Dinh T, Sajeev D, Kaya A, Topper E, Tan W, Memili E (2020). Cellular and functional physiopathology of bull sperm with altered sperm freezability. Front. Vet. Sci.

[54] Höfner L, Luther A.M, Palladini A, Fröhlich T, Waberski D (2020). Tolerance of stored boar spermatozoa to autologous seminal plasma:A proteomic and lipidomic approach. Int. J. Mol. Sci.

[55] Saraswat M, Joenväärä S, Jain T, Tomar A.K, Sinha A, Singh S, Yadav S, Renkonen R (2017). Human spermatozoa quantitative proteomic signature classifies normo-and asthenozoospermia. Mol. Cell Proteomics.

[56] Zhu W, Cheng X, Ren C, Chen J, Zhang Y, Chen Y, Jia X, Wang S, Sun Z, Zhang R, Zhang Z (2020). Proteomic characterization and comparison of ram (*Ovis aries*) and buck (*Capra hircus*) spermatozoa proteome using a data-independent acquisition mass spectrometry (DIA-MS) approach. PLoS One.

[57] Saadeldin I.M, Khalil W.A, Alharbi M.G, Lee S.H (2020). The current trends in using nanoparticles, liposomes, and exosomes for semen cryopreservation. Animals.

[58] Agossou D.J, Koluman N (2018). An objective analysis of factors affecting buck semen quality attributes during cryopreservation:A mini-review. Annu. Res. Rev. Biol..

[59] Wen F, Li Y, Feng T, Du Y, Ren F, Zhang L, Han N, Ma S, Li F, Wang P, Hu J (2019). Grape seed procyanidin extract (GSPE) improves goat semen quality when preserved at 4 C. Animals.

[60] Ahmed S, Khan M.I, Ahmad M, Iqbal S (2018). Effect of age on lipid peroxidation of fresh and frozen-thawed semen of Nili-Ravi buffalo bulls. Ital. J. Anim. Sci..

[61] Han Y, Peñagaricano F (2016). Unravelling the genomic architecture of bull fertility in Holstein cattle. BMC Genet..

[62] Jahanbin R, Yazdanshenas P, Rahimi M, Hajarizadeh A, Tvrda E, Nazari S.A, Mohammadi-Sangcheshmeh A, Ghanem N (2021). *In vivo* and *in vitro* evaluation of bull semen processed with zinc (Zn) nanoparticles. Biol. Trace Elem. Res..

[63] Majzoub A, Agarwal A (2018). Systematic review of antioxidant types and doses in male infertility:Benefits on semen parameters, advanced sperm function, assisted reproduction and live-birth rate. Arab J. Urol..

[64] Majzoub A, Agarwal A, Esteves S.C (2017). Antioxidants for elevated sperm DNA fragmentation:A mini-review. Transl. Androl. Urol..

[65] Dcunha R, Hussein R.S, Ananda H, Kumari S, Adiga S.K, Kannan N, Zhao Y, Kalthur G (2020). Current insights and latest updates in sperm motility and associated applications in assisted reproduction. Reprod. Sci.

[66] Silva D.M, Zangeronimo M.G, Murgas L.D, Rocha L.G, Chaves B.R, Pereira B.A, Cunha E.C (2011). Addition of IGF-I to storage-cooled boar semen and its effect on sperm quality. Growth Horm. IGF Res..

[67] Kumar A, Prasad J.K, Srivastava N, Ghosh S.K (2019). Strategies to minimize various stress-related freeze-thaw damages during conventional cryopreservation of mammalian spermatozoa. Biopreserv. Biobank..

[68] Bertero E, Maack C (2018). Calcium signaling and reactive oxygen species in mitochondria. Circ. Res..

[69] El-Battawy K.A (2019). Preservation of goat semen at 5°C with emphasis on its freezability and the impact of melatonin. Int. J. Vet. Sci. Res.

[70] Martin-Hidalgo D, Bragado M.J, Batista A.R, Oliveira P.F, Alves M.G (2019). Antioxidants and male fertility:From molecular studies to clinical evidence. Antioxidants.

[71] Poudel S.B, Dixit M, Neginskaya M, Nagaraj K, Pavlov E, Werner H, Yakar S (2020). Effects of GH/IGF on the aging mitochondria. Cells.

[72] Aguirre G.A, De Ita J.R, de la Garza R.G, Castilla-Cortazar I (2016). Insulin-like growth factor-1 deficiency and metabolic syndrome. J. Transl. Med.

[73] Pereira R, Sá R, Barros A, Sousa M (2017). Major regulatory mechanisms involved in sperm motility. Asian J. Androl.

[74] Silva D.M, Holden S, Souza J.C, Fair S (2019). *In vitro* addition of DHA and IGF-I increases the progressive motility of cryopreserved stallion semen. Rev. Agro Geoambiental.

[75] Bubenickova F, Postlerova P, Simonik O, Sirohi J, Sichtar J (2020). Effect of seminal plasma protein fractions on stallion sperm cryopreservation. Int. J. Mol. Sci..

[76] Dalal J, Kumar P, Chandolia R.K, Pawaria S, Rajendran R, Sheoran S, Andonissamy J, Kumar D (2019). A new role for RU486 (mifepristone):It protects sperm from premature capacitation during cryopreservation in buffalo. Sci. Rep.

[77] Syifa N, Yang J.T, Wu C.S, Lin M.H, Wu W.L, Lai C.W, Ku S.H, Liang S.Y, Hung Y.C, Chou C.T, Wang C.S, Ishihama Y, Liao J.H, Wu S.H, Wu T.H (2020). Phosphoproteomics and bioinformatics analyses reveal key roles of GSK-3 and AKAP4 in mouse sperm capacitation. Int. J. Mol. Sci..

[78] Betarelli R.P, Rocco M, Yeste M, Fernández-Novell J.M, Placci A, Azevedo Pereira B, Castillo-Martín M, Estrada E, Peña A, Zangeronimo M.G, Rodríguez-Gil J.E (2018). The achievement of boar sperm *in vitro* capacitation is related to an increase of disrupted disulphide bonds and intracellular reactive oxygen species levels. Andrology.

[79] Peris-Frau P, Martín-Maestro A, Iniesta-Cuerda M, Sánchez-Ajofrín I, Mateos-Hernández L, Garde J.J, Villar M, Soler A.J (2019). Freezing-thawing procedures remodel the proteome of ram sperm before and after *in vitro* capacitation. Int. J. Mol. Sci..

[80] Soma S, Jindal S.K, Kharche S.D (2016). Antioxidant and sperm:A complex story-a review. Indian J. Anim. Sci.

[81] Oliveira Resende C, Pedroso Betarelli R, Rabelo S.S, Resende Chaves B, Rodriguez-Gil J.E, Zangeronimo M.G (2019). Addition of insulin-like growth factor I (IGF-I) and reduced glutathione (GSH) to cryopreserved boar semen. Anim. Reprod. Sci..

[82] Van Hemelrijck M, Shanmugalingam T, Bosco C, Wulaningsih W, Rohrmann S (2015). The association between circulating IGF1, IGFBP3, and calcium:Results from NHANES III. Endocr. Connect..

[83] Nagata M.B, Egashira J, Katafuchi N, Endo K, Ogata K, Yamanaka K, Yamanouchi T, Matsuda H, Hashiyada Y, Yamashita K (2019). Bovine sperm selection procedure prior to cryopreservation for improvement of post-thawed semen quality and fertility. J. Anim. Sci. Biotechnol..

[84] Reis L.S, Ramos A.A, Camargos A.S, Oba E (2016). Integrity of the plasma membrane, the acrosomal membrane, and the mitochondrial membrane potential of sperm in Nelore bulls from puberty to sexual maturity. Arq. Bras. Med. Vet. Zootec..

[85] Wagner H, Cheng J.W, Ko E.Y (2018). Role of reactive oxygen species in male infertility:An updated review of the literature. Arab J. Urol.

[86] Peris-Frau P, Martín-Maestro A, Iniesta-Cuerda M, Sánchez-Ajofrín I, Cesari A, Garde J.J, Villar M, Soler A.J (2020). Cryopreservation of ram sperm alters the dynamic changes associated with *in vitro* capacitation. Theriogenology.

[87] Jin S.K, Yang W.X, Yang W.X (2017). Factors and pathways involved in capacitation:How are they regulated?. Oncotarget.

[88] Görlach A, Bertram K, Hudecova S, Krizanova O (2015). Calcium and ROS:A mutual interplay. Redox Biol..

